# Temperament and School Readiness – A Literature Review

**DOI:** 10.3389/fpsyg.2021.599411

**Published:** 2021-05-20

**Authors:** Petra Potmesilova, Milon Potmesil

**Affiliations:** ^1^Department of Christian Education, Sts Cyril and Methodius Faculty of Theology, Palacký University, Olomouc, Czechia; ^2^The Center of Evidence-Based Education and Arts Therapies, Faculty of Education, Palacký University, Olomouc, Czechia

**Keywords:** school readiness, temperament, self-control, preschool age, school success, effortful control

## Abstract

This review study was conducted to describe how temperament is related to school readiness. The basic research question was whether there is any relationship between later school success and temperament in children and, if so, what characterizes it. A systematic search of databases and journals identified 27 papers that met the two criteria: temperament and school readiness. The analytical strategy followed the PRISMA method. The research confirmed the direct relationship between temperament and school readiness. There is a statistically significant relationship between temperament and school readiness. Both positive and negative emotionality influence behavior (especially concentration), which is reflected in the approach to learning and school success.

## Introduction

Temperament, as a cluster of mental attributes that are presented in the form of experiencing and reacting to stimuli with an effect on emotional expressions and behavior, has an effect on school results amongst children (Keogh, 2003). For school education, therefore, what is important is how the child is able to manage its temperament and project it into activity, perseverance, and balance in response to stimuli (McClelland and Wanless, 2012).

The aim of this review study was to identify the relationship between the temperament of the child and school readiness presented in the scientific literature and how the research activities were constructed.

The definitions of temperament are not uniform in their conception and differ with different authors. Three basic theories have been put forward in relation to temperament in human life during its historical development: physiological theories Hippocrates or Galen (Ashton, 2013), bio-ecological theories (e.g., Thomas and Chess, 1977), and behaviorally oriented theories (e.g., Thomas and Chess, 1977). In the context of temperament research, current studies indicate terms that refine temperament and its manifestations, such as executive functions, effortful control, and self-regulation. Two basic research questions were identified in the context of the objective.

1. Are there studies that describe the relationship between temperament and school readiness and subsequent success rates in children?
2. If so, how can this relationship be characterized?

## Theoretical Background

### Temperament

Temperament is the focus of scientists’ interest in psychology. Perhaps the most prevalent are theoretical approaches to temperament as defined by Buss and Plomin (1975), Thomas and Chess (1977), Rothbart and Derryberry (1981), Goldsmith and Campos (1982), and Kagan (1984).

The Kagan approach (Kagan, 1984) is constructed based on biological factors that he considered congenital and may affect behavior. Goldsmith and Campos (1982) provide a definition of temperament as an individual difference in the ability to experience and express primal emotions. Differences in temperament are observable in the intensity of behavioral expressions, facial expressions, gestures, and movements. The definition, which is constructed on the basis of nine dimensions of behavioral styles – activity level, regularity, approach withdrawal, adaptability, threshold of responsiveness, intensity of reaction, quality of mood, attention span/persistence, and distractibility – was used by Thomas and Chess (as cited in Pharis, 1978). The model that was designed by Buss and Plomin (1975) was behavior-genetics oriented. It is assumed that early manifestations of temperamental features are hereditary and adapt evolutionally in a child, as responses to its living conditions, and are also relatively stable. Three core dimensions were identified: emotionality (E), activity (A), and sociability (S). The above-mentioned authors represent the primary sources to which most later studies relate. The approach to temperament by Rothbart (Rothbart and Derryberry, 1981) defines temperament as biologically ingrained individual differences in reactivity and self-regulation in emotional, activation, and attention-based processes. Reactivity refers to levels of biological arousal caused by changes in internal and external stimulation, which are captured as dimensions of negative influence and surgency. Self-regulation applies to processes that modulate reactivity and are reflected in a temperamental dimension that requires effortful control.

Temperament is accompanied by relatively permanent individual differences in reactivity and self-control that can be influenced in the course of the child’s development by maturation and experience (Rothbart and Bates, 1998). Differences in temperament are apparent from early childhood, with some children tending toward negativity and bad moods, while others have difficulties adapting to a new environment and people (Thomas et al., 1963; Putnam and Rothbart, 2006).

Children’s temperament has been described as a source of multiple categories of behavioral manifestations. The result is the concept of temperament as a three-component structure, which is represented by Surgency/Extraversion, Negative Affectivity, and Effortful Control (Rothbart, 1988; Rothbart and Bates, 1998, 2006; Rothbart and Putnam, 2002). In a more detailed concept, the Surgency/Extraversion category is described as impulsive, exhibiting a high degree of activity and courage and, at the same time, a need for satisfaction.

Negative Affectivity is characterized by manifestations of sadness, frustration, and being difficult to calm down. Effortful Control is characterized by the need for control and ability to concentrate (Rothbart and Putnam, 2002). In relation to school readiness and the subsequent success of children, Negative Affectivity is characterized by the above-mentioned authors as a possible source of problems with controlling emotions and thus as a possible source of problems in children’s behavior.

### Executive Functions

Executive functions as a term can be described as a collective name for a complex and diverse set of mental processes, the content and scope of which are differently defined. Most often, higher-order cognitive abilities are described using this term, allowing people to use psychological and physical resources effectively in an unknown or under-structured situation. Executive functioning, cognitive functioning, and affectivity can be considered as three fundamental dimensions of human behavior. Executive functions provide “know-how” on how to handle cognitive and affective processes. There is empirical evidence suggesting a strong relationship between temperamental characteristics and executive functions (Sudikoff et al., 2015). Affrunti and Woodruff-Borden (2015) state that the expression of temperament can be influenced by executive functioning. Temperament also includes behavioral aspects, as well as attention-seeking processes, including maintaining orientation and executive control. These skills form the basis for the development of self-regulation (Rothbart and Hwang, 2002).

### Effortful Control

The interaction of effortful control and emotion or stress is characterized by Zelazo et al. (2016) using the expressions “hot” effortful control and “cool” effortful control. These are based on the results of behavioral and neuroimaging research. Both types of effortful control are involved in the problem-solving function and varying degrees of motivation and emotion. For a “hot” approach, important situations involve the predominance of motivation and emotion. The “cool” approach works in affectively neutral contexts (Zelazo and Carlson, 2012).

### Self-Regulation

The current theoretical basis emphasizes the importance of self-regulation in relation to school readiness. Self-regulation in a broader sense involves the ability to control emotions (Blair and Raver, 2015). Self-regulation offers an important addition to the conceptualization of school readiness because it addresses children’s ability to attend to information, use it appropriately, and inhibit behavior that interferes with learning. However, like the broader concept of school readiness, theories and perspectives on self-regulation have focused on various priorities (Pan et al., 2019).

The level of reactivity is related to the characteristics of the reactions to changes in stimuli that are reflected on several levels (behavioral, autonomous, and neuroendocrine) and display different periods of observable parameters from latency and an increase and then a peak of intensity until relaxation. Self-control influences these processes and influences reactivity (Rothbart et al., 2004).

### School Readiness

School readiness is understood as the state when a child enters school adequately prepared to engage in school activities and benefit from the educational situations so that he/she can experience success regarding his/her potential. Kagan (1990) speaks about readiness for learning, which is a state in which the child, thanks to his/her development, is able to learn the individual subjects. Janus (2007) describe school readiness as a level of maturity of the nervous system which allows the child to process specific “school” stimuli and develop his/her skills and knowledge without mental suffering.

Regarding mental development, school readiness is a child’s state when the child’s skills necessary for meeting his/her cognitive, physical, and social needs on entry to school can be employed (Mashburn and Pianta, 2006; Pianta et al., 2007; Janus and Gaskin, 2013). The developmental level of the child provides the opportunity to safely reflect the needs of schooling in a wider context in terms of cognitive, social, and emotional functions (Lemelin et al., 2007).

In relation to the above, one can also include maturity and physical health, emotional maturity, and the necessary communication skills (Kagan, 1992; Doherty, 2007).

Janus and Offord (2000) named the basic domains that are important in relation to a child’s functioning at school, which can at the same time be used as areas for evaluation or in the event of a need for diagnostics of particular functions. These are physical health and well-being, including the necessary development of fine and coarse motor skills. It is also a domain that includes the social skills of responsibility and respect, approach to education, and readiness to explore new things. Attention also needs to be paid to emotional maturity, which includes pro-social behavior and the ability to function in a group. Being able to deal with anxiety and fear and the ability to manage one’s behavior regarding concentration and activity are associated with emotional maturity. According to these authors, the other domains on the list are the level of language skills and the overall level of cognitive functioning in the areas of literacy, mathematical imagination, and motivation to learn. Communication skills and their adequate development as an essential factor for effective schoolwork can be emphasized.

## Methods

The research scope of the study is focused on the school readiness of children in relation to their temperament. The given age category of the children and their temperament are considered essential with regard to their readiness for, and subsequent success in, school education, as is stated by other expert studies. Vágnerová (2012) considers preschool age to be a period during which the child should be mentally and physically sufficiently mature to begin school attendance, while Al-Hendawi (2013) argues that temperament is a significant parameter of school adaptation and success. Al-Hendawi (2013) also states that the authors of expert studies view temperament from different perspectives.

The aim of the research was to determine whether there are studies that deal with the relationship between temperament, its dimensions, and school readiness.

For this review study, a design was applied that is based on the PRISMA method (Moher et al., 2015) in the context of the theory of Paré and Kitsiou (2017). Four stages of the work process were created based on this method.

### Stage 1– Strategy

The study, and therefore the search for the primary source texts, focused on the period from 1 January 2000 to 29 February 2020, with the selection including articles in scientific journals in English. The search keywords were represented by the following expressions: School readiness; Temperament; Preschool age; School success; Effortful control; Self control; Mood.

The following elements were used for the search strategy: (school N1 readiness) OR (school N1 success); (school N1 readiness) OR (school N1 success) AND mood; (school N1 readiness) OR (school N1 success) AND Effortful control; (school AND readiness) OR (school AND success); (school AND readiness) OR (school AND success) AND Effortful control; (school AND readiness) OR (school AND success) AND mood; (school N/3 readiness) OR (school N/3 success) AND mood AND preschool.

This time span was chosen because the largest number of texts for further analysis was searched for in the databases during this period. The choice of a shorter time span of the margin did not offer sufficient saturation in searching.

### Stage 2 – The Selection of Databases for the Search

The MEDLINE, CINAHL, ERIC, EMBASE, PsycINFO, PsycArticles, Web of Science, Google Scholar, Scopus, and Proquest databases were used for the search. The EBSCO Discovery Service was used. A total of 1092 articles were found.

### Stage 3

**FIGURE 1:**
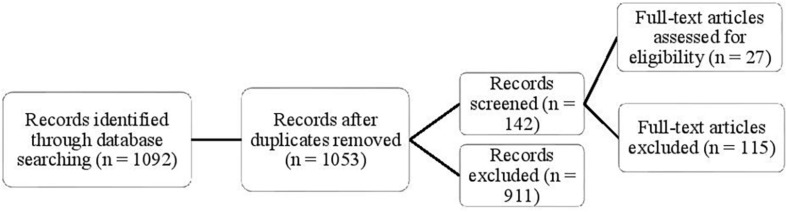
Flowchart of Searching.

Abstracts were analyzed for all 1092 articles. On the basis of this analysis, those articles that did not match the specified criteria were gradually eliminated. Figure 1 shows what the procedure for the selection of suitable articles looked like.

In the last stage a detailed analysis of 142 articles was performed. In all these articles, the key categories “Temperament”, “Executive functions” “Effortful control”, “Self-regulation”, and “School readiness” were used.

On the basis of the analysis of 142 articles, specific groups based on the topics were created. School readiness was related to different variables with an indirect relationship to temperament – ADHD (25 articles), autism (one article), illness and health problems (19 articles), different age categories (28 articles), a conflict between the parents’ and teachers’ expectations of preschool-age children (five articles), and the topic of preschool children and disability (one article). In addition, there was the theory of mind and executive functions (eight articles), language skills (two articles), and the environment of the family and school (eight articles), parents’ temperament (nine articles), and teacher temperament (nine articles).

The narrow selection included 26 or 27 articles whose topics matched the requirements of the relationship between school readiness and temperament, i.e., both the essential categories – school readiness and temperament – appeared in them simultaneously. Only the 27th article (Miller and Goldsmith, 2017) is rather specific because the authors wanted to create an ideal pupil who would be successful at school.

The articles were analyzed qualitatively using a set of qualitative indicators. The indicators were determined in compliance with the research questions as the basis for the research and a more detailed description of the relationship between the child’s temperament and school readiness. On the basis of these criteria, three qualitative indicators were determined: methods, target group, and research results. These indicators were then divided into the sub-groups shown in Table 1.

**TABLE 1:** Qualitative indicators.

**METHODS**	**TARGET GROUP**	**CONCLUSION**
1. What was observed	1. Number	1. Confirmation of the relationship
2. Method of data collection	2. Gender	2. Risk
3. Complementary method	3. Age period	3. Protection
4. Design	4. Who responded	4. Notes
5. Definition of temperament	5. Ethnicity	
	6. Specifics	

The stated qualitative indicators were determined as the basis for further examination and a more detailed description of the relationship between the child’s temperament and school readiness or success in the selected articles.

## Results

### Qualitative Indicator – Methods

The focus of the selected studies was divided into three fundamental domains: temperament (A), cognitive abilities (B), and social skills (C) (see Table 2). In twelve studies (Schoen and Nagle, 1994; Rudasill and Konold, 2008; Rudasill and Rimm-Kaufman, 2009; Stacks and Oshio, 2009; Zhou et al., 2010; Gartstein et al., 2016; Collings et al., 2017; Miller and Goldsmith, 2017; VanSchyndel et al., 2017; Bryce et al., 2018; Beceren and Özdemir, 2019; Johnson et al., 2019) the authors directly use the term ‘temperament’, while in 15 (Bramlett et al., 2000; Valiente et al., 2008, 2010; Rimm-Kaufman et al., 2009; Iyer et al., 2010; Rhoades et al., 2011; Silva, 2011; Valiente et al., 2011; Willoughby et al., 2011; Al-Hendawi and Reed, 2012; Razza et al., 2012; Morris et al., 2013; Gaias et al., 2016; Sawyer et al., 2019; Fung et al., 2020) they use the term ‘regulation of emotions’, which they perceive as part of temperament. In all the research focused on school readiness, however, the concept of readiness differed, and it was possible to divide it into two basic categories of social skills (Bramlett et al., 2000; Rimm-Kaufman et al., 2009; Rudasill and Rimm-Kaufman, 2009; Stacks and Oshio, 2009; Valiente et al., 2010; Zhou et al., 2010; Silva, 2011; Valiente et al., 2011; Willoughby et al., 2011; Al-Hendawi and Reed, 2012; Morris et al., 2013; Gaias et al., 2016; Gartstein et al., 2016; VanSchyndel et al., 2017; Johnson et al., 2019; Beceren and Özdemir, 2019) and cognitive skills (Schoen and Nagle, 1994; Rhoades et al., 2011; Valiente et al., 2011; Razza et al., 2012; Morris et al., 2013; Collings et al., 2017; Miller and Goldsmith, 2017; Bryce et al., 2018; Johnson et al., 2019; Sawyer et al., 2019; Rimm-Kaufman et al., 2009; Valiente et al., 2010; Zhou et al., 2010; Willoughby et al., 2011; Gaias et al., 2016; Gartstein et al., 2016; VanSchyndel et al., 2017). In the area of cognitive skills, the authors observed reading and mathematical concepts (Valiente et al., 2010; Morris et al., 2013; Gaias et al., 2016; Johnson et al., 2019), language skills (Schoen and Nagle, 1994; Rhoades et al., 2011), and in two cases both the skills (Razza et al., 2012; Sawyer et al., 2019).

**TABLE 2:** Qualitative indicator – target group.

**Art.**	**Number**	**Gender %**		**Age period**	**Who responded**	**Ethnicity**	**Specifics**
		**♂**	**♀**				
1	104	Not stated		1st year of primary school	Parents and teachers	98% Caucasian, 2% minority	Not stated
2	77	54.5	45.5	5-11 years	Parents and teachers	74% Afro-Americans, 16.9% Caucasian, 6.5% Hispanics, and 2.6% other ethnicity	Specific requirements in education resulting from increased risks of adverse circumstances (economic disadvantage, developmental delay, combination of both)
3	324	52	48	4-7 years	Parents and trained professionals	74% Afro-Americans, 16.9% Caucasian, 6.5% Hispanics and 2.6% other ethnicity	87% of children included in the free lunch program
4	241	52	45	Ø 5.44	Parents	78% of Mexican/Mexican-American ethnic origin, 8% were non-Latino Caucasian, 7% identified as other, 6% of the children were African-American, and 1% were Native American	Children in the Head Start program
5	74	33.8	66.2	36-68 months	Parents	55.4% of the children Caucasian, 17.6% Afro-Americans, 20.3% mixed ethnicity, and 6.8% unclassified	Children in the Head Start program
6	10,700	Not stated		Preschool age	Teachers	39.29% Caucasian, 21.16% Afro-Americans, 33.66% Hispanics, 5.9% Asians	Children in these types of programs: “Head Start” and “pre-K” (pre-kindergarten).
7	152	40	60	Ø 72 months	Teachers	Not stated	Average economic situation
8	2595	52	48	5 years	Trained professionals	21.9% Caucasian, 52.1% Afro-Americans, 23.1% Hispanics, 2.9% other	76% of the children were born to single mothers
9	3410	51	49	0-7 years	Teachers	Australian – representative sample	Representative sample
10	341	47	53	Ø 4.5	Parents and teachers	69% Afro-Americans, 18% Multi-racial, 12% Hispanics, 1% Caucasian	Children in the Head Start program
11	1364	705	659	4.5	parents, teachers	1097 white	Representative sample
12	74	41	33	5–6 years	teachers	60.8% white, 9.5% black, 14.9% Latino, 6.8% Asian, 4.1% multiracial, 6.2% other	kindergarten children from primarily low-income families
13	214	118	96	T1 55-97m	parents, children, teachers	77%; 80%; 78% Caucasian, 12%; 12%; 11% Hispanic, 5% others	6-year longitudinal study, 2-year period for T1., T2, and T3 milestones; Family SES, and especially income as a robust predictor of achievement.
	193	105	88	T2 2 years after			
	159	88	71	T3 4 years after			
14	390	212	178	6-10 years	teachers, children, peers,	38.2% Latino, 46.7% white, 15.1% other races,	Low- and middle-income families
15	264	122	142	7-12 years	parents, children, teachers	52% Mex-Am., 34% Eur-Am., 8% Afr-Am., 6% Native Am.,	Representative sample
16	819	406	413	54 months and 1st grade of school	parents, teachers	84% Caucasian, 10% Black or Afr-Am, 6% others.	Representative sample
17	172	92	80	4.70-6.24 years	teachers,	83.7% Caucasian, 13.4% Afr – Am., 2,9% others	Rural children
18	829	not stated	not stated	3-5 years	parents, teachers,	Percentage not stated: Afr- Am., Euro-Am., Hispanic and others.	Time span of 2 years; I. 2006 II. 2007. Private preschools as well as public Head Start centers participated. Free lunches for 60%.
19	926	50%	50%	3-5 years	researchers, teachers,	58% Afr-Am., 31% Caucasian, 10% Hispanic, 1% another racial group,	Head Start 50% children, 50% community childcare,
20	425	44,5%	55,5%	6.6-9.1 years	parents, teachers, children,	Chinese children	Families with low SES
	382	47,1%	52.9%	10.1-12.9 years			
21	114	57%	43%	18 months, 42 -54 months	parents, teachers,	82.4% non-Hispanic, 83.1% Caucasian	Representative sample
22	291	58%	42%	avg. 67.72 months	parents, teachers,	70% White, 14% Latino, 8% Asian, 3% Black, <1% Am- Ind.	Students attended regular education classrooms in public schools in the southwestern United States.
23	174	49%	51%	M = 6.48	teachers; children	Caucasian 60% Hispanic/Latino 29% Asian 5% African American 2% Other/mixed ethnicity 4%	Urban children
24	31	18	13	4 months longitudinally until 4 years	parents,	Caucasian 92.3%	All participants were healthy, typically developing children, no specifics regarding economic status, single parenting, specific educational support.
25	284	137	174	60 months	teachers, parents	Turkish (not stated exactly)	Representative sample
26	523	52.9%	47.1%	52.42 months	teachers, parents	Hong Kong children	Representative sample
27	29 teachers	Not stated		4 years	Teachers	Not stated	Teachers “generate” the profile of the most successful child who enters school prepared the best

To characterize temperament, different tools were used, in eleven cases the CBQ questionnaire (Rudasill and Konold, 2008; Rudasill and Rimm-Kaufman, 2009; Iyer et al., 2010; Valiente et al., 2010; Zhou et al., 2010; Silva, 2011; Valiente et al., 2011; Morris et al., 2013; Gaias et al., 2016; Miller and Goldsmith, 2017; Bryce et al., 2018), which will also be used in our case. In order to assess the level of cognitive and social skills, certified tools were mainly used, in one case (Johnson et al., 2019) a tool that the researchers developed themselves, and in two cases, observation was used (Rimm-Kaufman et al., 2009; Rudasill and Rimm-Kaufman, 2009).

The definition of temperament is then adapted for the purpose of the studies. In eight cases, the authors put an emphasis on individual differences in their definitions (Bramlett et al., 2000; Rudasill and Konold, 2008; Rudasill and Rimm-Kaufman, 2009; Valiente et al., 2010; Gartstein et al., 2016; Collings et al., 2017; Bryce et al., 2018; Johnson et al., 2019), in eleven cases they emphasized self-control (Rimm-Kaufman et al., 2009; Valiente et al., 2010, 2011; Willoughby et al., 2011; Gaias et al., 2016; Gartstein et al., 2016; Collings et al., 2017; Miller and Goldsmith, 2017; Bryce et al., 2018; Johnson et al., 2019; Sawyer et al., 2019), and in five cases they stressed the biological basis (Bramlett et al., 2000; Rudasill and Konold, 2008; Rudasill and Rimm-Kaufman, 2009; Al-Hendawi and Reed, 2012; Sawyer et al., 2019). Morris et al. (2013), Beceren and Özdemir (2019), Johnson et al. (2019), and Fung et al. (2020) stress the influence of temperament on emotions in their definition and the influence on children’s social skills is emphasized in nine studies (Schoen and Nagle, 1994; Valiente et al., 2008; Stacks and Oshio, 2009; Iyer et al., 2010; Zhou et al., 2010; Rhoades et al., 2011; Silva, 2011; Razza et al., 2012; VanSchyndel et al., 2017).

### Qualitative Indicator – Target Group

The numbers of respondents were representative in relation to the research that was analyzed. In longitudinal studies, there were research studies with large numbers of respondents (more than 1000) (Razza et al., 2012; Johnson et al., 2019; Sawyer et al., 2019), but also one research study involving 31 respondents (Gartstein et al., 2016). For most other research studies, the number of respondents ranged between 100 and 1000 (Schoen and Nagle, 1994; Bramlett et al., 2000; Valiente et al., 2008, 2010, 2011; Rimm-Kaufman et al., 2009; Rudasill and Rimm-Kaufman, 2009; Iyer et al., 2010; Zhou et al., 2010; Rhoades et al., 2011; Silva, 2011; Willoughby et al., 2011; Gaias et al., 2016; Collings et al., 2017; VanSchyndel et al., 2017; Bryce et al., 2018; Beceren and Özdemir, 2019; Fung et al., 2020). The exceptions consisted of some studies (Stacks and Oshio, 2009; Al-Hendawi and Reed, 2012; Morris et al., 2013) in which there were fewer than 100 respondents and one case with 1364 respondents (Rudasill and Konold, 2008). In one study (Miller and Goldsmith, 2017) the respondents were teachers whose task was to create basic categories which they could use to assess a child’s school readiness.

In four cases (Bramlett et al., 2000; Silva, 2011; Miller and Goldsmith, 2017; Johnson et al., 2019) the authors of the study do not state the results regarding gender. In the studies by Schoen and Nagle (1994), Stacks and Oshio (2009), Valiente et al. (2010), and VanSchyndel et al. (2017) the gender ratio between boys and girls was 40% to 60% and in the remaining studies the ratio was around 50% in all cases.

The age span of the respondents was between 0 and 12 years of age. The age of the respondents was associated with the research aim (see Table 2 and the glossary accompanying the table). The information about the respondents was in all cases (except in one case, Gartstein et al., 2016), obtained from the responses of teachers or trained researchers and in 14 cases (Bramlett et al., 2000; Rudasill and Konold, 2008; Valiente et al., 2008, 2010, 2011; Rudasill and Rimm-Kaufman, 2009; Zhou et al., 2010; Rhoades et al., 2011; Silva, 2011; Al-Hendawi and Reed, 2012; Collings et al., 2017; VanSchyndel et al., 2017; Beceren and Özdemir, 2019; Fung et al., 2020) also from parents. In three cases, information was also obtained from children (Iyer et al., 2010; Zhou et al., 2010; Valiente et al., 2011).

Schoen and Nagle (1994), Miller and Goldsmith (2017), and Beceren and Özdemir (2019) do not state ethnicity in their studies. Sawyer et al. (2019) state that the research was carried out on a representative sample of the Australian population, similarly to Bramlett et al. (2000), who state that 98% of their sample was Caucasian. In the case of these two studies, the aim was not to compare the influence of temperament on school success with regard to ethnicity, but primarily a description of the given relationship in a representative sample of the given population. Silva (2011) cites ethnicity, but not the percentual distribution. Rudasill and Konold (2008), Zhou et al. (2010), and Fung et al. (2020) presented mono-ethnic samples; in the first case they were Caucasians, the second study involved children from Hong Kong, and in the third article the respondents were from China. In the other studies the percentages of the ethnic groups are presented.

Schoen and Nagle (1994), Bramlett et al. (2000), Rudasill and Konold (2008), Rudasill and Rimm-Kaufman (2009), Valiente et al. (2010), Gartstein et al. (2016), Beceren and Özdemir (2019), Sawyer et al. (2019), and Fung et al. (2020) do not state any specifics in relation to their respondents or state that it was a representative sample. Miller and Goldsmith (2017) aimed their research at creating a profile of the most successful child who enters school prepared to the maximum extent. Rimm-Kaufman et al. (2009) reported that their respondents were exclusively children from villages, while in contrast Gaias et al. (2016) chose children from cities. In other cases, the authors studied children who came from a socially or economically endangered environment. They were specifically children who were born to single mothers (Razza et al., 2012), children who were included in the “Head Start” program (Stacks and Oshio, 2009; Rhoades et al., 2011; Silva, 2011; Willoughby et al., 2011; Bryce et al., 2018; Johnson et al., 2019), and children who were included in the free lunch program (Silva, 2011; Collings et al., 2017). Iyer et al. (2010), Zhou et al. (2010), Valiente et al. (2011), Al-Hendawi and Reed (2012), and Morris et al. (2013) were interested in children who displayed specific requirements for education as a result of increased risk of adverse circumstances (economic disadvantage, developmental delay, or a combination of both).

### Qualitative Indicator – Conclusion

In the case of the study by Bryce et al. (2018), it was not possible to confirm a hypothetical chain process: child’s positive emotionality → emotional engagement in kindergarten → behavioral expressions in kindergarten → educational results in kindergarten. In other cases, the link between temperament and school readiness or subsequent school success was confirmed.

In some cases (Rudasill and Konold, 2008; Valiente et al., 2008, 2010, 2011; Rudasill and Rimm-Kaufman, 2009; Iyer et al., 2010; Zhou et al., 2010; Rhoades et al., 2011; Silva, 2011; Al-Hendawi and Reed, 2012; Morris et al., 2013; Gaias et al., 2016; Gartstein et al., 2016; Collings et al., 2017; Miller and Goldsmith, 2017; VanSchyndel et al., 2017; Bryce et al., 2018; Beceren and Özdemir, 2019; Johnson et al., 2019; Fung et al., 2020) the authors were further interested in whether temperament can be seen as a risk or protective factor. In most cases, it was found that higher Effortful Control has a positive relationship to greater school readiness – the success rate and lower Effortful Control can predict behavioral problems and thus problems at school (Valiente et al., 2008, 2010, 2011; Iyer et al., 2010; Zhou et al., 2010; Morris et al., 2013; Gartstein et al., 2016; VanSchyndel et al., 2017). Rudasill and Rimm-Kaufman (2009), Silva (2011), and Gaias et al. (2016) add that the value of Effortful Control can influence the teacher’s relationship with the child and thus the child’s school readiness and also later school success. Al-Hendawi and Reed (2012) found that negative emotionality has a significant effect on adaptivity and schoolwork and can become a predictor of inappropriate behavior. In contrast, Johnson et al. (2019) did not confirm that problems in the area of a child’s temperament can be perceived as a significant predictor of prosocial behavior. There is a statistically significant relationship between temperament and school readiness. Both positive and negative emotionality influence behavior (especially concentration), which is reflected in the approach to learning and school success.

Collings et al. (2017) suggest that there was a positive effect of a previous intervention on temperament, confirmed in the individual items of school performance. Their results for the boys who participated in the intervention program were better in the areas of literacy and mathematics than was the case in boys who did not participate. Bryce et al. (2018) state that positive emotionality significantly influenced behavior in children in kindergarten. Rudasill and Konold (2008), Rhoades et al. (2011), Beceren and Özdemir (2019), and Fung et al. (2020) characterized the child’s maturity in the context of how he/she is able to control his/her temperament so that it can function as a supportive factor in education. Similar conclusions were also reached by Miller and Goldsmith (2017). In their view, children who were able to regulate their emotions were able to react better in socially appropriate ways and focus their attention, which facilitates learning and provides higher chances of success in school education.

In addition, difficult temperament at an early age can lead to low parental involvement at age three. The role of difficult temperament, poor maternal involvement, and externalizing behavior may be partially responsible for the continuity that has been observed in antisocial behavior over time (Walters, 2014).

The last thing that the authors state is the more detailed characteristics of the relationship identified between temperament and school readiness or school success. Bramlett et al. (2000) admit that there might be differences between what can be termed the home and school temperaments, which can explain the differences between the parents’ and children’s answers. Beceren and Özdemir (2019) stress the importance to social-emotional adjustment of family involvement. Schoen and Nagle (1994), Al-Hendawi and Reed (2012), Collings et al. (2017), and Miller and Goldsmith (2017) state that here there are differences between the temperaments of boys and girls; the last two argue that boys show higher activity. Razza et al. (2012), Collings et al. (2017), and also Gartstein et al. (2016) suggest that there is a positive effect of intervention programs on school readiness. These are programs that focus on exerting control over one’s temperament during preschool age. Similarly, Sawyer et al. (2019) state that if there is an increase in the ability to exert self-control at the ages of 2-3 and 6-7, this can have a positive influence on school readiness. The ability to self-regulate is considered an essential factor in school readiness by Rudasill and Konold (2008), Valiente et al. (2010), Willoughby et al. (2011), and VanSchyndel et al. (2017), and Valiente et al. (2008), Zhou et al. (2010), Valiente et al. (2011), Morris et al. (2013), and Fung et al. (2020) attribute great importance to effortful control for school readiness. Another important factor that can affect a child’s school readiness is his/her relationships with peers (Iyer et al., 2010) and teachers (Rudasill and Rimm-Kaufman, 2009; Silva, 2011; Gaias et al., 2016). Rimm-Kaufman et al. (2009) state that the quality of the preschool classroom affects the child’s behavior, and this can then affect school readiness. Miller and Goldsmith (2017) argue that the model of “an ideal child” was created separately for boys and girls who will be successful at school.

### Summary

The analysis of literary sources showed that in the period under consideration, there are expert studies dealing with the relationship between temperament and school readiness. In total 27 articles were included in the narrowest selection, in which the authors sought and examined this relationship or perceived it as the default setting for further examination.

From selected studies it is clear that when working with the phenomenon of temperament, as a factor that can influence other phenomena from the point of view of psychology, there is a big problem with the definition of temperament. In the introduction to the rewiev study, the individual definitions and views of their authors on temperament are given. The following are terms that are used by other authors instead of temperament. Table 3 lists the concepts of temperament as presented by the authors of selected 27 studies. In the analyzed studies, the authors used either the term temperament or the concept of regulation of emotions directly. Temperament or regulation of emotions were then characterized from different points of view using terms: self-control, individual differences, biological basis and social skills. These concepts of temperament in selected articles confirm the high degree of difference of approaches to the concept of temperament.

**TABLE 3:** Qualitative indicator – methods.

**Art.**	**What was observed^a^**			**Design^b^**	**Definition of temperament**
	**A**	**B**	**C**		
1	+	−	+	1	Individual differences in the tendency of behavior with the onset in childhood and relative stability over the further course of life (Orth and Martin, 1994).
2	+	−	+	1	The authors define temperament on the basis of several current theories from which they abstract three common constructions for temperament: 1) biological fundamentals; 2) possible identification already at an early age; 3) apparent more as a tendency in behavior.
3	+	+	−	1	Temperament is described by the authors according to studies by Eisenberg et al. (2000), Rothbart et al. (2000), Derryberry and Reed (2002), Cole et al. (2004), and Zentner and Shiner (2012) as a source of specific features of the ability to possess self-control in a child.
4	+	+	−	1	The authors define temperament as inborn individual differences in reactivity and the ability to display self-control (Rothbart et al., 2004, p. 357).
5	+	−	+	1	The authors approach temperament as part of the complex of a child’s behavioral expressions with an effect on his/her social skills, which are also influenced by the level of attachment (Belsky and Fearon, 2002; Spieker et al., 2003).
6	+	+	+	1	Temperament is perceived as a multidimensional construct, which is individually different in terms of the ability to exert self-control especially in the areas of reactivity, emotions, and attention (Rothbart and Bates, 1998).
7	+	+	−	1	The authors of the study use the characteristics of temperament as a predictor of the manner or style of the social and physical interaction of the child with the environment.
8	+	+	−	1	The authors work with the assumption that temperament in childhood is one of the factors influencing the intentional attention of the child.
9	+	+	−	1	The authors of the study work with the knowledge that temperament, attention, and the ability to manage emotional expressions are identified as characteristics that have a biological basis and are relatively stable over the course of childhood (Thomas et al., 1963).
10	+	+	−	1	Understanding emotions is regarded as a crucial aspect of social awareness, which is one of the complexes of socio-emotional skills in a receptive and expressive form. One of the essential components is temperament, which, together with cognitive and other functions, influences the quality of the child’s school readiness and later his/her results in education.
11	+	-	−	1	Temperament is an individual’s biologically based, multidimensional (e.g., emotionality, activity level, shyness, effortful control) style of responding to the environment (Thomas and Chess, 1977).
12	+	+	+	1	Effortful control is defined as a child’s ability to utilize attentional resources and to inhibit behavioral responses in order to regulate emotions and related behaviors (Rothbart and Ahadi, 1994).
13	+	+	+	1; 3	Effort control is a group of temperamentally based skills viewed as the basis of self-regulation (Rothbart and Bates, 2006). EC is the efficiency of executive attention.
14	+	−	−	1	Effortful control skills represent such competencies as could account for both children’s risk of peer victimization and poor school-related outcomes.
15	+	−	−	1	Effortful control was used as an index of children’s regulatory abilities: “the efficiency of executive attention—including the ability to inhibit a dominant response and/or to activate a subdominant response, to plan, and to detect errors”
16	+	−	+	3	Temperament is an individual’s general style of responding to stimuli in the environment. It is a biologically based, multi-dimensional construct that begins to emerge during infancy and childhood, is molded by environmental forces, and provides the foundation for personality traits in older children, young people, and adults (Thomas and Chess, 1977; Kagan and Fox, 2006; Rothbart and Bates, 2006).
17	+	+	+	3	An important dimension of temperament is effortful control, the broad construct of self-regulation that incorporates a set of related skills involving emotion, attention, behavior, and cognition.
18	+	−	+	1	Effortful control, the regulatory aspect of temperament, has been defined as “the efficiency of executive attention, including the ability to inhibit a dominant response and/or to activate a subdominant response, to plan, and to detect errors” (Rothbart and Bates, 2006, p. 129).
19	+	+	+	1	Self-regulation as one of the major achievements of early childhood refers to the process through which children increasingly acquire the ability to regulate their own arousal, emotion, and behavior (Kopp, 1982; Shonkoff and Phillips, 2000).
20	+	+	+	1	Effortful control and anger/frustration are temperament characteristics which are associated with a wide range of adjustment outcomes in children and adolescents, including behavioral problems, social competence, and moral and conscience development (Eisenberg and Morris, 2002; Rothbart and Bates, 2006). As a multidimensional construct including various capacities such as the voluntary focusing of attention (e.g., concentrate when studying) and suppressing inappropriate responses (Derryberry and Rothbart, 1997; Rothbart and Bates, 2006).
21	+	+	+	1	Childhood temperament is hypothesized to drive social and personality development throughout the lifespan (Rothbart and Ahadi, 1994).
22	+	+	+	1	Temperament is “constitutionally based individual differences in reactivity and self-regulation, in the domains of affect, activity, and attention” (Rothbart and Bates, 2006).
23	+	+	+	1	Effortful control is a predictor of adaptive functioning across developmental domains in early schooling, defined as “the ability to inhibit a dominant response to perform a subdominant response and/or to activate a subdominant response, to plan, and to detect errors”, a set of temperamentally based skills that form the basis of self-regulation.
24	+	+	+	1	Temperament is constitutionally based individual differences in reactivity and self-regulation in the domains of affect, activity, and attention (Rothbart and Bates, 2006). Structurally, temperament in childhood has been defined in terms of three major domains: Negative Emotionality, Positive Affectivity/Surgency, and Extraversion (Putnam et al., 2001; Gartstein and Rothbart, 2003).
25	+	−	+	1	Temperament is emotionally motivating and shaped by human experience and adaptive variations (Derryberry and Rothbart, 1997). Temperament as the psychological source of genetics in a person, a psychic aspect of DNA (Beceren and Özdemir, 2019).
26	+	−	−	1	Children’s emotional regulation depends on their temperamental regulation or effortful control (Rothbart and Bates, 2006; Eisenberg et al., 2000).
27	+	+	−	3	The authors work with the definition of temperament as individual differences in behavioral reactivity and the ability to manage, which are directly linked to socio-emotional and communicative skills (Goldsmith and Harman, 1994).

*^*a*^What was observed: A = temperament, B = cognitive ability, C = social skills.*

*^*b*^Design: quantitative = 1, qualitative = 2, mixed = 3.*

Out of 27 relevant studies, 26 confirmed a statistically significant relationship between temperament and school readiness; see Table 4. In one case (Bryce et al., 2018), the authors did not confirm the relationship between temperament and school readiness, but at the same time they stated that the results support the hypothesis about the indirect influence of positive emotional adjustment in the child on his/her behavior and afterwards on his/her school results. The results of the selected studies indicate that there are differences between boys and girls in the area of temperament, which is then reflected in the level of school readiness; see Table 2. We should therefore consider this fact in the child-raising/educational process. Another thing that needs to be taken into account in the educational process is the relationship between children’s temperament and the temperament of teachers. This relationship can have an impact on school readiness and success at school. Apart from the confirmation of the relationship between temperament and school readiness, the authors of the studies also dealt with the description of this relationship. The authors agree that the inability to manage one’s emotions has a significant influence on one’s behavior, such as the ability to concentrate or intentional attention, and afterwards one’s readiness for school. If an individual is able to manage his/her emotions, he/she is able to react in a socially appropriate manner and is able to focus, and this can facilitate his/her learning, which is a prerequisite for school success.

**TABLE 4:** Qualitative indicator – conclusions.

**Art.**	**Notes**
1	Possible differences between home and school temperament.
2	Boys showed a higher level of activity, impulsiveness, and emotional intensity, and a lower level of shyness. Girls showed a higher level of attempts at self-control and a higher level of social skills and adaptivity. Girls showed cooperative behavior, more partner sympathy, and a more positive attitude to school. The authors speak about possible greater tolerance for some “negative” behavior in boys than in girls.
3	The influence of gender, temperament, and the children’s subsequent participation in specific programs while of preschool age on school work.
4	The results tend to support the thesis about the indirect influence of a child’s positive emotional tune and behavioral expressions influenced by that and afterwards his/her results at work.
5	These results are also in compliance with the results of other studies by Martin et al. (1988) and also Howse et al. (2003).
6	Not stated.
7	All the TABC scales of assessed temperament were significantly associated with a pre-reading score. Furthermore, the study showed that the boys in the group were more active but lost concentration more easily, and in their behavior and in their reactions in the class they were more emotional.
8	The authors do not demonstrate the influence of the socio-economic background of the family, maternal warmth, or difficult expressions of temperament on school success in the sample of children. The authors emphasize the need for intervention during preschool age in children who show difficulties in controlling their temperament to foster real prevention of difficulties at the beginning of education.
9	The results show that children whose task attentiveness increases between the ages of 2 and 3 and 6 and 7 show better results in literacy and in mathematical imagination than children whose results in task attentiveness are worse in the given period. Similarly, it concerns the area of self-control of emotions. Children whose ability in emotional regulation at the ages of 2-3 and 6-7 increases show better results in literacy during school attendance.
10	The results of this study also suggest that regardless of demographic criteria, the functioning of intentional attention is one of the essential elements in the school success of a child. The results of the study support the statement of the mutual influence of emotional relationship, intentional attention, and results in education.
11	Inhibitory control and attentional focusing (i.e., effortful control) contributed to teachers’ ratings of children’s social competence. Children with high levels of inhibitory control and attentional focusing were rated higher on cooperation and self-control. Effortful control is denied as the ability to inhibit an inappropriate response and activate an appropriate one. Students who are highly cooperative and show high levels of self-control are doing just that.
12	Effortful control strongly correlates with school readiness and achievement among kindergarteners. The effects of effortful control were not affected (moderated) by demographic variables. No matter of children’s sex and household income children with high effortful control demonstrated better school readiness, math and reading skills.
13	Effortful control was positively related to social functioning, and social functioning was positively related to achievement, even when SES, age, and sex were used as covariates (i.e., as predictors of academic achievement).
14	The emotional experience of being bullied undermines children’s ability to engage effectively in classroom activities by interfering with their effortful control functioning. Peer victimization correlated negatively with effortful control at each time point, and effortful control was predictive of school engagement and academic achievement.
15	There is evidence that academic competence is associated with effortful control and children’s relationships, but it is not clear if effortful control provides unique prediction of academic competence or if relationships partially mediate the effortful control and academic competence associations.
16	The relationship between child- and teacher-initiated interactions in the context of effortful control and lower levels of effortful control predicted more frequent teacher-initiated interactions. Teachers interacted more frequently with children low in effortful control to provide reminders concerning behavior and attention, and these interactions may be viewed negatively by children as restrictive in their nature.
17	Classroom quality did not moderate the relation between children’s attributes and engagement in school. The classroom quality is important in relation to children’s adaptive classroom behaviors but protective in other unmeasured areas, such as self-directedness or planfulness, which involve more sophisticated forms of self-regulation, such as metacognition and the development of motivational styles.
18	With increased concerns about children’s school readiness there has been a focus on improving academic skills and the quality of teachers’ instructional styles. Teachers should be aware that early conflictual relationships may have long-term consequences for how children feel about school and that conflict with some children may be more likely and have an impact to their school success.
29	Self-regulatory tasks were strongly correlated with child academic outcomes.
20	Children displaying temperament precursors (e.g., low effortful control) to academic problems may be identified as early as beginning school age. These children can benefit from interventions that target the cognitive, interpersonal, and motivational processes associated with low effortful control and school failure.
21	Children who are well-regulated and impulsive may have an advantage in terms of academic achievement. Matching between impulsivity and approach emotions may also be advantageous for achievement in early childhood.
22	Students who are able to regulate their emotions in the classroom have a distinct advantage over their less-regulated peers. Effortful control is likely to influence academics as children progress through school.
23	Learning about and reflecting on students’ and teachers’ own temperamental characteristics—can help these functions in concert; teachers may become more aware of how attributes such as effortful control shape their classroom practices and interactions with students.
24	The relation of infant temperament in the context of the emergence of basic knowledge/pre-academic skills holds promise for applications relying on temperament to screen children at risk of difficulties at school entry, and possibly to identify those most likely to benefit from interventions.
25	Social emotional adjustment by temperament and empathy; the subdimensions of temperament significantly predicted the social emotional adjustment subdimensions of family involvement, social confidence, readiness for school, and emotional adjustment.
26	There is a utility to supporting kindergarten children’s readiness for school to foster their future emotional regulation and because of that to reduce potential problems.
27	In total, five clusters were created according to their connection to the characteristics of school readiness in the group of children who were observed. The evaluation showed no differences between the boys and girls. A model of “an ideal child” for boys and girls was created; they show a high level of positive approach, excitement about work, endurance, curiosity, the necessary social skills, and a tendency to cooperate. The hypothetical child showed a minimal level of negativism and disturbing reactions.

In 14 out of the 27 cases, there were respondents from a socio-economically disadvantaged environment; see Table 2. The authors do not confirm the direct influence of a socio-economic disadvantage on school readiness or success but characterize the temperament of these children in relation to searching for appropriate upbringing and educational procedures. They also show the success of these procedures, which does not comply, however, with the theories of temperament, which are based on the fact that temperament is inborn and relatively stable (e.g., Orth and Martin, 1994).

In searching for specialized texts focused on the relationship of temperament and school readiness, we repeatedly encountered the concept of the relationship of temperament to cognitive functions. Specifically, temperament is part of effortful control directly related to executive attention (Rothbart et al., 2007). Frick et al. (2018) described the relationship between temperament and cognitive function in their research. Their work focuses on cognitive self-regulation as a set of constructive behaviors that influence cognitive abilities to integrate learning processes. These processes are planned and customized to support the tracking of personal goals in a changing environment. This function already develops when the child is at an early age. When the child is of school age, temperament is associated with cognitive abilities. With regard to the part of the study by Chong et al. (2019) in which they focused on preschool age, the authors report that temperament was less related to cognitive and academic outcomes after parenting and family confusion had been taken into account.

Temperament is considered a predictor of functional attention influenced by individual differences in reactivity and self-regulation in emotion and activity (Rothbart et al., 2006; similarly, Guarnera et al., 2019). Outside the topic of research, but as a critical problem area, there appears the relationship of temperament (especially its projection into the attention) and learning difficulties and the connection with the possibility of special intervention (Commodari, 2012). Gan et al. (2016) draw attention to the possible influence of the environment (rural – city) on temperament and subsequently on children’s school readiness. The quality of the teacher-child relationship or direct teacher intervention can have a positive influence on the relation between emotional regulation and cognitive skills (Commodari, 2013; Guarnera et al., 2017). The relationship of individual components of temperament and cognitive function in school-age children – especially reading, writing, and mathematics – is evidenced in their study (Guarnera et al., 2017).

## Discussion and Possible Application in Practice

By analyzing the selected articles, the basis for creating answers to the key questions was obtained.

1. There is a significant relation between temperament and its major dimensions and school readiness.
2. Temperament and its dimensions can affect school success in both directions, positively and negatively.

Children whose Effortful Control is the dominant feature can be assumed to possess the ability to exert control and self-regulate in the field of behavior (Olson et al., 2005).

If the level of Surgency/Extraversion is higher in the context of the child’s behavior, it can be considered a risk factor that affects hyperactivity. To a lesser extent, it can be an inhibitor of the “research approach” but irrespective of the school’s instructions and rules. The presence of the above options can be a source of problems in children’s behavior and thus have a negative effect on school readiness outcomes (Fox et al., 2001). However, manifestations of children’s behavior, as an important element of school readiness, are always the result of the relationship between temperament and its interaction with the environment. For more on this see Rothbart and Putnam (2002).

School attendance and the child’s subsequent success in education can be influenced by more factors. The major factors that experts (Janus, 2007; Merrell and Tymms, 2007; Vágnerová, 2012) list include cognitive functions, motivation, experience, and the child’s temperament. Temperament can influence a child’s functioning during school performance and therefore to some extent either enhance or limit the child’s performance. In mathematics, reading, or other school activities which require the child to calm down, concentrate on the task, and resist stimuli from the surroundings, temperament can be a very important factor (Collings et al., 2017; Ato et al., 2020). Therefore, it can have a negative influence on the performance of a child who is functioning cognitively quite well, but is unable to concentrate, calm down, and detach him- or herself from disturbing stimuli from outside. On the other hand, it can enhance a child’s performance, which might be weaker from the school evaluation perspective. They can, to an adequate extent, reduce their physical activity, calm down, concentrate, and carry out a task to its end.

Al-Hendawi and Reed (2012) argue that the negative emotionality associated with a low level of ability to control expressions of temperament can be a source of problems in social situations in class. In their study, these authors point out the possibility of the overstimulation of children with stimuli from the outside, with a negative effect on their engagement in schoolwork and the quality of their results. Dependency in the teacher-child relationship has a strong correlation with school adjustment difficulties, including poorer academic performance, more negative attitudes to school, and less positive engagement with the school environment (Birch and Ladd, 1997).

In terms of temperament and its introduction into the school environment, there is one potentially conflicting area (Keogh and Prokopcová, 2007). These are situations where the child’s temperament and the temperament of the teacher do not meet in a mutually satisfactory constellation, but are mismatched with each other, creating clashes and having a negative effect on their mutual functioning.

The quality of first-grade classroom environments is based on three domains: emotional support, classroom organization, and instructional support. A high-quality classroom environment may ameliorate the academic and social risks associated with having a difficult temperament (Curby et al., 2011).

Some teachers are active and react quickly, while some are slower and react upon consideration. These differences are reflected in the activities which take place in the classroom, especially in the pace of teaching and in the form of personal interactions and emotional charge. If there is a child in the group with a significantly different temperament to that of the teacher, this difference may be a source of misunderstandings and consequently of failure and demotivation in the child. The child will experience more stressful situations when entering school. Apart from the encounter with the teacher’s temperament, there is also the encounter with the temperaments of the child’s classmates. If it is important to deal with temperament and success at school, it is not on the basis of a construct, but on the actual situation in each classroom and the need to work effectively with these factors. In conclusion, it should be noted regarding the school or class environment that they appear explicitly in only two articles as one of the parameters linked to the temperament of children. In the first case, Al-Hendawi and Reed (2012) are inclined to the concept of the school environment in terms of the creation and functioning of social relations. They work with relationships between children and children and the teacher. In the second article, Bramlett et al. (2000) used the term ‘school environment’ for the social environment and focused on the area of problematic behavior, which is related to the reduced ability of the child to control his or her temperament.

The preschool period of the child is a very important period in which the basics of socio-emotional competence are laid. Their influence on future success in education and in the development of socialization is indisputable. Teachers can use specific programs – such as Head Start or their own active approach – to help children successfully develop self-regulatory behavioral control skills and thus help prepare them for school success (McBryde et al., 2004; McClelland and Wanless, 2012; Brophy-Herb et al., 2018; Booth et al., 2019). In conclusion, the authors cited above agree on temperament as an innate individual reactivity to stimuli that can affect the school success rate of children.

The analysis of the articles also showed that even if the temperament is innate, it can be affected by appropriate interventions, so that it can be used in a positive direction in school success. Methodologically, this study will be used to process a similar study that will focus on the areas of children with visual handicaps.

## Weaknesses

The focus on texts written in English can thus be a weakness. It is possible that this topic might be covered in other languages, but the results of such studies are not presented here. The authors are aware of possible terminological differences that can occur in the texts, as was the case, for example, with the term ‘temperament’, for which some authors used the term ‘mood’.

## Author Contributions

PP and MP contributed to the design and implementation of the review, to the analysis of the results, and to the writing of the manuscript. All authors contributed to the article and approved the submitted version.

## Conflict of Interest

The authors declare that the research was conducted in the absence of any commercial or financial relationships that could be construed as a potential conflict of interest.
